# Molecular, Metabolic and Physiological Responses to Boron Stress in Higher Plants

**DOI:** 10.3390/plants12112136

**Published:** 2023-05-28

**Authors:** María Reguera, Juan José Camacho-Cristóbal

**Affiliations:** 1 Departamento de Biología, Universidad Autónoma de Madrid, c/Darwin 2, Campus de Cantoblanco, 28049 Madrid, Spain; 2 Departamento de Fisiología, Anatomía y Biología Celular, Facultad de Ciencias Experimentales, Universidad Pablo de Olavide, 41013 Sevilla, Spain

## 1. Background

Although the essentiality of boron (B) for plant growth has recently been questioned [1], raising an exciting discussion between “Boronists” [2,3], its requirement in plant development was first established at the beginning of the 20th century [4]. B, as boric acid (H_3_BO_3_) and tetrahydroxyl borate anion B(OH)_4_^−^, can form complexes with biological compounds containing two hydroxyl groups in *cis*-configuration. In this regard, it has been proposed that the binding capacity of B plays a key role in biological systems by stabilizing molecules with *cis*-diol groups, regardless of the role played by these molecules [5]. Nonetheless, to date, the only evidence for a direct involvement of B in plant metabolism comes from its structural role in the cell wall through the formation of ester bonds between the borate anion and the apiose residues of two molecules of rhamnogalacturonan II (RGII) [6,7]. However, this evidence cannot explain the plethora of plant processes affected by both B deficiency and toxicity, so the possibility that other primary functions of B may exist should not be ruled out. Furthermore, it should be noted that B is an essential element for vascular plants that have a very narrow range of optimal concentrations, so controlling its availability to plants in soils and irrigation water is crucial for agricultural production. In fact, both B deficiency and toxicity have negative effects on the development and yield of crop plants [8,9,10].

Therefore, knowledge of the molecular, metabolic, and physiological changes induced by B deficiency or toxicity, as well as the characterization of the signaling pathways for these responses, which has advanced greatly in recent years, can contribute not only to a better understanding of the role of B in plants, but can also provide efficient strategies to improve B stress tolerance in plants. In line with this, this Special Issue of *Plants* presents a collection of eight manuscripts covering basic and applied topics on the response of vascular plants to B stress.

## 2. Main Findings Considered in this Special Issue

### 2.1. One-Time Foliar Application and Continuous Resupply via Roots Equally Improved the Growth and Physiological Response of B-Deficient Oilseed Rape [11]

B deficiency problems usually occur in regions with high rainfall conditions where boric acid in the soil solution can easily leach out. The symptoms of B deficiency in crop plants cultivated on B-deficient soils can be alleviated by the application of B fertilizers, as soil fertilization or foliar spray. In this regard, Dinh et al. [11] have studied the effectiveness of B resupply via roots and leaves to alleviate several symptoms of B deficiency in oilseed rape (*Brassica napus* L.) plants grown hydroponically. They concluded that both types of B fertilization to oilseed rape plants can partially resolve the effects of B deficiency on dry matter production, net photosynthesis, and sugar contents, and on the expression of specific B transporters.

### 2.2. Crosstalk of Cytokinin with Ethylene and Auxin for Cell Elongation Inhibition and Boron Transport in Arabidopsis Primary Root under Boron Deficiency [12]

The knowledge on the signaling pathways for the response of plants to B deficiency has advanced greatly in recent years. For instance, recent studies revealed the involvement of hormones (including ethylene, auxin, cytokinin, brassinosteroids, and ABA), calcium, and reactive oxygen species in the orchestrated signaling pathways of B stress responses [13]. Findings in these fields can contribute not only to a better understanding of the role of B in plants, but also provide efficient strategies to improve B stress tolerance in plants. In this regard, Herrera-Rodríguez et al. [12] provided evidence that cytokinins negatively regulate root cell elongation in Arabidopsis plants subjected to B deficiency through two independent pathways involving ethylene and auxins. The results also suggest that these phytohormones regulate the gene expression of several B transporters in the root.

### 2.3. Salt-Pretreatment-Mediated Alleviation of Boron Toxicity in Safflower Cultivars: Growth, Boron Accumulation, Photochemical Activities, and Antioxidant Defense Response [14]

B toxicity and salinity stresses often occur together, so there is growing interest in studying the interactive effects of these stresses on plant growth [15]. The work of Arslan et al. [14] analyzed the potential use of salt pretreatment to alleviate the effects of B toxicity on growth, phytoremediation capacity, photosynthesis, and oxidative stress in two safflower cultivars (*Carthamus tinctorius* L.). Results suggested that salt pretreatment partially mitigated several of the biochemical and physiological changes caused by B toxicity.

### 2.4. Carbon-11 Radiotracing Reveals Physiological and Metabolic Responses of Maize Grown under Different Regimes of Boron Treatment [16]

B deficiency can alter plant growth and physiology, which has been related to its role in stabilizing cell wall structure [17]. In this sense, monocots have generally shown a lower B requirement, which has been linked to a reduced pectin content compared to dicotyledonous plants. However, positive responses in terms of plant productivity have been reported in different cereal species, including maize [18]. In the work performed by Wilder et al. [16], the impact of B on carbon partitioning was evaluated using ^11^C-radiotracing, highlighting the strong effect of B deficiency on metabolism. Furthermore, they demonstrated that synergistic and antagonistic relations occur among certain mineral nutrients when changing B supply.

### 2.5. Citrus Physiological and Molecular Response to Boron Stresses [19]

B is an essential element for vascular plants that has a very narrow range of optimal concentrations, so controlling its availability to plants in soils and irrigation water is crucial for agricultural production. In fact, both B deficiency and toxicity have negative effects on the development and yield of crop plants [8,9,10]. In this regard, the essentiality of B in citrus plants was discovered soon after B was shown to be essential for plants [4,20], and since then different works have described the effects of B deficiency or toxicity in these plant species. Yang et al. [19] reviews the research performed in Citrus when subjected to B stress, and emphasizes the importance of further analyzing B transport and allocation in Citrus, a genus that comprises important crops for the fruit industry.

### 2.6. Silicon Differently Affects the Apoplastic Binding of Excess Boron in Wheat and Sunflower Leaves [21]

B toxicity problems often occur in crops of arid/semiarid regions with low rainfall and high water evaporation where B accumulates in the uppermost layers of the soil, reaching toxic levels for plants. Alleviating the symptoms of B toxicity in crop plants can be achieved by applying elements that have a positive impact on the physiological tolerance of plants (i.e., those preventing oxidative stress). For instance, Savic et al. [21] demonstrated that Si fertilization significantly decreased B concentration in wheat (*Triticum vulgare* L.) and sunflower (*Helianthus annuus* L.) leaves when growing under high-B conditions, leading to a recovery of plant growth, especially in wheat. Interestingly, Si application increased the B-binding capacity of the leaf apoplast in wheat but not in sunflower.

### 2.7. Response of Maize (Zea mays L.) to Drought under Salinity and Boron Stress in the Atacama Desert [22]

Drought can interfere with B influx as it limits water transpiration and uptake [23]. On the other hand, interactions between salinity and B stress (that could act simultaneously, especially in arid and semiarid regions [24]) have been described in different plant species and linked to limited water uptake [25]. The work of Riveros-Burgos et al. [22] evaluated the effect of the combination of the three stressors (drought, salinity, and high B) on the physiological response of ‘lluteño’ maize (*Zea mays* L.), which is adapted to the Atacama Desert, pointing to a genotypic-dependent response in maize to the simultaneous action of these environmental stresses.

### 2.8. What Can Boron Deficiency Symptoms Tell Us about Its Function and Regulation? [26]

B-deficient plants may exhibit a wide range of symptoms depending on the species and the developmental stage of the plant [27]. In recent years, the use of novel molecular tools has allowed for the discovery of novel functions and ligands of B in organisms including bacteria, animals, and plants. Still, there is a general lack of knowledge regarding the specific functions of B, B stress signaling pathways, and B interactors beyond its structural role in the plant cell wall. Bolaños et al. [26] proposed a model integrating B-deficiency symptoms and signaling pathways, summarizing the current knowledge regarding B putative ligands in plants.

## Data Availability

Data sharing not applicable.
